# Multiple Merger Genealogies in Outbreaks of *Mycobacterium tuberculosis*

**DOI:** 10.1093/molbev/msaa179

**Published:** 2020-07-15

**Authors:** Fabrizio Menardo, Sébastien Gagneux, Fabian Freund

**Affiliations:** 1 Department of Medical Parasitology and Infection Biology, Swiss Tropical and Public Health Institute, Basel, Switzerland; 2 University of Basel, Basel, Switzerland; 3 Department of Plant Biodiversity and Breeding Informatics, Institute of Plant Breeding, Seed Science and Population Genetics, University of Hohenheim, Stuttgart, Germany

**Keywords:** *Mycobacterium tuberculosis*, demographic inference, multiple-merger coalescents, approximate Bayesian computation, random forest

## Abstract

The Kingman coalescent and its developments are often considered among the most important advances in population genetics of the last decades. Demographic inference based on coalescent theory has been used to reconstruct the population dynamics and evolutionary history of several species, including *Mycobacterium tuberculosis* (MTB), an important human pathogen causing tuberculosis. One key assumption of the Kingman coalescent is that the number of descendants of different individuals does not vary strongly, and violating this assumption could lead to severe biases caused by model misspecification. Individual lineages of MTB are expected to vary strongly in reproductive success because 1) MTB is potentially under constant selection due to the pressure of the host immune system and of antibiotic treatment, 2) MTB undergoes repeated population bottlenecks when it transmits from one host to the next, and 3) some hosts show much higher transmission rates compared with the average (superspreaders).

Here, we used an approximate Bayesian computation approach to test whether multiple-merger coalescents (MMC), a class of models that allow for large variation in reproductive success among lineages, are more appropriate models to study MTB populations. We considered 11 publicly available whole-genome sequence data sets sampled from local MTB populations and outbreaks and found that MMC had a better fit compared with the Kingman coalescent for 10 of the 11 data sets. These results indicate that the null model for analyzing MTB outbreaks should be reassessed and that past findings based on the Kingman coalescent need to be revisited.

## Introduction

The coalescent is a stochastic mathematical model that formally describes the shapes of the expected genealogies in a population (Kingman 1982b). The original formulation of Kingman has been extended to include different evolutionary processes, such as fluctuations in population size (Griffiths and Tavaré 1994), population subdivision and migration (Wilkinson-Herbots 1998), recombination (Hudson 1983), and selection (Kaplan et al. 1988; Neuhauser and Krone 1997).

Although the genealogy of a sample is typically unknown, mutational models can be superimposed onto the coalescent to describe DNA sequence polymorphisms. These are generally easy to obtain from natural populations, thus opening the possibility of data-based statistical inference.

Applications of the coalescent include the study of the evolutionary histories and population dynamics of a variety of taxa (Kuhner 2009), including humans (Excoffier et al. 2013) and pathogens (Pybus et al. 2001; Joy et al. 2003), and the identification of genetic loci under selection (Biswas and Akey 2006; Hernandez et al. 2011).

One of the assumptions of the Kingman coalescent is that the variation in reproductive success among individuals is sufficiently small (e.g., the variance of offspring per individual is bounded regardless of population size), such as at most one pair of sampled lineages can find a common ancestor for any single time point on the coalescent time scale.

This assumption is relaxed in a more general class of models, the so-called multiple-merger coalescents (MMC). MMC have been developed to model scenarios in which the variance in reproductive success is large enough to cause the coalescence of more than two lineages at the same time point on the coalescent time scale (Möhle and Sagitov 2001; see Tellier and Lemaire [2014] for a review). Some of the underlying discrete generation models leading to MMC allow for very large offspring numbers of one or more individuals in a single generation (Schweinsberg 2003; Eldon and Wakeley 2006). However, MMC genealogies can also arise if one individual has many descendants in a relatively small number of generations, so that this family leads to multiple mergers after collapsing discrete generations to arrive at the timescale of the continuous time coalescent. In this article, we will refer to “skewed offspring distribution” to indicate variation in reproductive success that leads to multiple merger genealogies. For details on how coalescent models arise from discrete generation models, we refer to the relevant mathematical literature (Kingman 1982a; Griffiths and Tavaré 1994; Möhle and Sagitov 2001; Freund 2020).

Compared with the Kingman coalescent, MMC have been proposed to be more appropriate models to investigate marine organisms with sweepstakes reproduction (Sargsyan and Wakeley 2008), agricultural pathogens with recurrent seasonal bottlenecks (Tellier and Lemaire 2014), loci under positive selection (Durrett and Schweinsberg 2005), and rapidly adapting pathogens (Neher and Hallatschek 2013).

Despite a growing interest in MMC, there are few studies that used genetic polymorphisms to test whether MMC are indeed better fitting models compared with the Kingman coalescent. Signatures of MMC have been detected at the *creatin kinase muscle type A* locus of the Atlantic cod (*Gadus morhua*; Árnason and Halldórsdóttir 2015), in the mitochondrial genome of Japanese sardines (*Sardinops melanostictus*; Niwa et al. 2016), in populations of breast cancer cells (Kato et al. 2017), and in the B-cell repertoire response to viruses such as HIV-1 and influenza (Horns et al. 2019; Nourmohammad et al. 2019). Although MMC are theoretically appealing genealogy models for pathogen samples (Irwin et al. 2016; Neher and Walczak 2018; Rocha 2018), their fit to observed data in pathogen populations has not been investigated so far. Only very recently, MMC have been used to study the within-host genetic diversity of *Mycobacterium tuberculosis* (MTB), a major human pathogen causing tuberculosis (Morales-Arce et al. 2020).

Here, we look for evidence of MMC in between-host populations of MTB*.* Between-host populations of MTB are expected to have a skewed offspring distribution because of three reasons: 1) MTB is an obligate pathogen, and therefore potentially constantly adapting under the pressure of the host immune system and of antibiotic treatment (Gagneux 2018); 2) superspreaders; these are patients responsible for a very large number of secondary infections compared with the average (Gardy et al. 2011; Walker et al. 2013; Ypma et al. 2013; Lee, Radomski, Proulx, Manry, et al. 2015; Stucki et al. 2015; Lee et al. 2020), thus causing a large variance of the pathogen’s offspring size; and 3) MTB undergoes repeated bottlenecks when transmitting from one host to the next, with a few bacteria, and potentially as few as one, founding the entire population infecting the new host (Lin et al. 2014).

Additionally, a low genetic diversity and an excess of rare variants (singletons) have been reported in MTB (Hershberg et al. 2008; Pepperell et al. 2013), and both are known signatures of MMC genealogies (Tellier and Lemaire 2014).

Methods based on the Kingman coalescent are often used in population genetic analyses of MTB; for example: 1) The Bayesian skyline plot (BSP, Drummond et al. 2005) has been used to infer past population dynamics in tuberculosis outbreaks, finding evidence for constant effective population size (Bainomugisa et al. 2018), rapid effective population growth (Eldholm et al. 2015; Folkvardsen et al. 2017; Brown et al. 2019), or slow effective population decline (Lee, Radomski, Proulx, Levade, et al. 2015); 2) different methods have been used to infer the demographic history of the global MTB population (Comas et al. 2013; Pepperell et al. 2013; Bos et al. 2014) and of individual MTB lineages (Wirth et al. 2008; Kay et al. 2015; Luo et al. 2015; Liu et al. 2018; Merker et al. 2015, 2018; Huang et al. 2019; Mulholland et al. 2019; O’Neill et al. 2019), finding evidence for effective population growth or for complex fluctuations that have been correlated with major events in human history such as the introduction of antibiotic treatment; and 3) the strength of purifying selection was estimated with a simulation-based approach, finding a genome-wide selection coefficient several order of magnitude higher compared with other prokaryotes and eukaryotes (Pepperell et al. 2013).

Although some of these results might be biased by unaccounted population structure (Heller et al. 2013) or sampling biases (Lapierre et al. 2016), potentially they are all impacted by the violation of the Kingman’s assumption described above, and their conclusions could be affected by model misspecification (Tellier and Lemaire 2014).

Given the undergoing efforts in controlling and stopping the spread of tuberculosis, and the global impact of this pathogen that causes more than 1.4 million deaths each year (WHO 2019), it is important to evaluate the adequacy of the population genetic models used to study tuberculosis epidemics. To this end, we considered 11 MTB whole-genome sequence (WGS) data sets, and used an approximate Bayesian computation (ABC) approach based on simulations to find the best-fitting model among Kingman’s coalescent, and two MMC models, the Beta coalescent (Schweinsberg 2003) and the Dirac coalescent (Eldon and Wakeley 2006). We found that MMC were the best-fitting model for 10 of the 11 data sets (nine fitted best to the Beta, one to the Dirac coalescent). In addition, we investigated the consequences of violating the assumption on the offspring distribution when performing demographic inference with the BSP and found that it leads to the inference of false population dynamics. Consequently, demographic inference based on models assuming nonskewed offspring distribution (i.e., Kingman’s coalescent) likely leads to inaccurate results when applied to MTB epidemics, and potentially to the epidemics of other pathogens with similar life histories.

## Results

### Models and Data Sets

MTB is thought to be strictly clonal, with lateral gene flow completely absent, or very rare (Hershberg et al. 2008; Gagneux 2018; Chiner-Oms et al. 2019). Therefore, the MTB genome can be considered as a single genetic locus, and one single genealogy describes the relationships among all MTB strains in any data set. The shape of the genealogy of a sample is influenced by many factors, such as the underlying offspring distribution, sampling scheme, population subdivision, geographic population structure, migration, and changes in population size. To avoid these confounding effects, we considered only populations that were unlikely to be affected by population structure, sampling biases, population subdivision, and migration. We searched the literature for WGS data sets of MTB where all strains were sampled from a single phylogenetic clade that was restricted to a particular geographic region, and identified 11 studies. Most of these data sets represent single outbreaks (Materials and Methods). For each data set, we downloaded the raw Illumina sequences (supplementary table 1, Supplementary Material online) and used a bioinformatic pipeline described in the Materials and Methods section to identify high-confidence single nucleotide polymorphisms (SNPs) (table 1). To test the robustness of our analyses to different SNP call procedures, we performed an additional SNP call altering one key parameter: the minimum proportion of reads supporting an SNP call (from 90% to 75%, see Materials and Methods). We found that the allele frequency spectrum (one of the most important statistics, see below) was robust to the different SNP call settings (supplementary figs. 1 and 2, Supplementary Material online). We performed the main analyses (see below) on both data set variants. As the results were similar, and we consider the SNP call with the 75% threshold less stringent, in the article we report the results for the data sets based on the 90% threshold. The results for the data sets based on the 75% threshold and the comparison between the two different sets are reported in supplementary table 2, Supplementary Material online.

**Table 1.** Data Sets Used in This Study.

Data Set^a^	Number of Strains	Number of Polymorphic Positions	Locality of Sampling
Eldholm 2015	248	497	Buenos Aires (Argentina)
Lee 2015	147	454	Nunavit (Canada)
Stucki 2016	175	6,264	Central African countries
Shitikov 2017	176	1,164	Russia and Belarus
Roetzer 2013	61	74	Hamburg (Germany)
Comas 2015	21	1,334	Ethiopia
Bainomugisa 2018	81	401	Daru Island (PNG)
Bjorn-Mortensen 2016	121	128	East Greenland
Folkvardsen 2017	702	214	Copenhagen (Denmark)
Stucki 2014	60	128	Bern (Switzerland)
Eldholm 2016	25	17	Oslo (Norway)

a We identified the data sets with the first author’s name and year of the original publication.

Excluding population structure, two factors that can shape the diversity of these data sets are changes in population size, and whether offspring distributions are skewed. We modeled changes in population size assuming exponential population growth, as has often been done in previous studies (Eldholm et al. 2015; Merker et al. 2015; Eldholm et al. 2016; O’Neill et al. 2019).

We modeled skewed offspring distributions with two MMC models deriving from explicit population models:

1. The Beta coalescent, in which the probability of each individual to coalesce in a multiple merger event is regulated by a Beta distribution with parameters α (between 1 and 2) and 2 − α. The Beta coalescent was originally introduced to model populations with sweepstakes reproduction (Schweinsberg 2003), but it was also proposed to capture the genealogies of populations undergoing recurrent bottlenecks and of epidemics characterized by superspreaders (Tellier and Lemaire 2014; Hoscheit and Pybus 2019). Lower values of α (closer to one) correspond to larger multiple merger events, and for α = 1 the Beta coalescent corresponds to the Bolthausen–Sznitman (BSZ) coalescent (Bolthausen and Sznitman 1998). The BSZ coalescent is an explicit model for genealogies of populations evolving under rapid positive selection, which lead certain families of selected genotypes to have strongly increased sizes compared with the average (Brunet and Derrida 2013; Neher and Hallatschek 2013).
2. The Dirac coalescent, also known as psi coalescent, is defined by a single parameter (ψ). The parameter ψ represents the average proportion of sampled lineages that coalesce in a single multiple merger event. The Dirac coalescent was derived from a modified Moran population model, where at each generation, with a small probability, a single individual produces a proportion ψ of the next generation, instead of just two offspring. This gives an alternative model with skewed offspring distribution (Eldon and Wakeley 2006).

Importantly, none of these MMC models was derived from a population model specific for MTB. Nevertheless, they are useful to investigate whether processes leading to skewed offspring distribution (on the coalescent time scale) are important in MTB, and we will discuss this further below.

In a first analysis, we tested whether modeling skewed offspring distributions alone explained the observed genetic diversity better than modeling variable population sizes (with an exponential growth model) and standard offspring distributions. Therefore, we considered MMC models with constant population sizes. It was previously shown that even for a single locus, these hypotheses can be distinguished for moderate sample sizes and high enough mutation rates (Eldon et al. 2015; Freund and Siri-Jégousse 2020).

Subsequently, we explored whether modeling skewed offspring distribution together with variable population size (exponential growth) further improved the fit to the data.

### Model Selection and Parameter Estimation with ABC

For model selection and parameter estimation, we used an ABC approach based on random forests (RF), as reported in detail in the Materials and Methods section. We considered four models: Kingman’s coalescent with constant population size (KM), Kingman’s coalescent with exponential population growth (KM+exp), Beta coalescent with constant population size (BETA), and Dirac coalescent with constant population size (Dirac). Briefly, for each data set, we collected the SNPs identified with the bioinformatic analysis, reconstructed the genotype of the most recent common ancestor (MRCA) and used it to polarize the SNPs. We then calculated a set of 24 summary statistics measuring genetic diversity and phylogenetic properties. For each model, we performed 125,000 simulations of a sample of size *n*, where *n* is the number of individuals in the data set, drawing the scaled mutation rate from a prior distribution spanning 1 order of magnitude around the Watterson estimator (θ_obs_). As described in Pudlo et al. (2016), we performed model selection via ABC using a random forest of 1,000 decision trees. For parameter estimation within a model class, we followed the approach of Raynal et al. (2019).

We found that for most data sets, the ABC approach had overall good discriminatory power, with out-of-bag (OOB) error rates (the misclassification probabilities, see Materials and Methods and table 2 for details) ranging from 4% to 16.4%. The only exception was the data set Eldholm 2016 (OOB error rate = 32.2%), which was the data set with the lowest genetic diversity. Most importantly for our study, the probability that data generated under a model with standard offspring distribution (KM and KM+exp) were misclassified as multiple merger was low (1.1–7%), again the only exception was the data set Eldholm 2016 (18%).

**Table 2.** Results of Model Selection and Parameter Estimation.

Data Set	Selected Model	OOB Error Rate (misclassification % as MMC)^a^	Posterior Probability	Median and 95% Posterior CI of Coalescent Parameters^b^
Eldholm 2015	BETA	5.4% (2.1%)	96.6%	α: 1.19 (1.01–1.39)
Lee 2015	BETA	6.8% (2.6%)	96.0%	α: 1.27 (1.06–1.59)
Stucki 2016	KM+exp	4.1% (1.1%)	100%	*g*: 2,828 (676–4,508)
Shitikov 2017	KM+exp	5.3% (1.8%)	99.7%	*g*: 2,833 (1,158–4,819)
Roetzer 2013	BETA	15.8% (7.0%)	95.7%	α: 1.23 (1.02–1.50)
Comas 2015	BETA	16.4% (5.4%)	87.3%	α: 1.31 (1.05–1.72)
Bainomugisa 2018	BETA	8.9% (3.3%)	97.6%	α: 1.22 (1.02–1.51)
Bjorn-Mortensen 2016	BETA	10.2% (4.2%)	80.6%	α: 1.03 (1.00–1.16)
Folkvardsen 2017	BETA	4.0% (1.4%)	97.8%	α: 1.13 (1.02–1.33)
Stucki 2015	KM+exp	13.2% (5.4%)	87.4%	*g*: 13,288 (3,756–19,932)
Eldholm 2016	Dirac	32.2% (18.0%)	77.1%	ψ: 0.36 (0.19–0.61)

a The OOB error rate is the probability that a simulation is misclassified as coming from any other model class, between parentheses we report the probability that a simulation generated with KM or KM+exp is misclassified as an MMC (BETA or Dirac).

b The interval between the 0.025 quantile and the 0.975 quantile of the parameter of the selected model (*g* for KM+exp, ψ for Dirac, and α for BETA). The growth rate *g* is reported as used in Hudson’s ms (for diploid genealogies; Hudson 2002), thus all growth estimates have to be halved to be interpreted for MTB.

We found that BETA was the best-fitting model for 7 of the 11 data sets, KM+exp was the best model for three data sets, and Dirac was the best model for one data set. For all but one data set (Eldholm 2016), the posterior probability of the selected model was higher than 80% and therefore more than four times more likely than all other models combined (table 2 and supplementary table 2, Supplementary Material online).

One potential problem when performing model selection is that none of the considered models is able to generate key features of the observed data (i.e., the considered models are not adequate; Chapter 6 in Gelman et al. 2013). To exclude this possibility, we performed posterior predictive checks, in which for each data set, we simulated data under the best-fitting model using the median of the posterior distribution of the relative parameter. We then compared the observed data with the simulated data. If the selected model is adequate, we expect the simulated and observed data to be similar. Conversely, if the selected model is not adequate, we expect simulated and observed data to be different. We found that for all but two data sets, the observed values of 20 summary statistics were within the range of values obtained from the simulated data, indicating that the best model can reproduce the features of the observed data (supplementary figs. 3–13, Supplementary Material online). The two exceptions were Stucki 2016 and Shitikov 2017, for which, respectively, the 0.9 quantile of the Hamming distance, and the mean of the minimal observable clade size statistic were not overlapping with the simulated values (supplementary figs. 12 and 13, Supplementary Material online). This indicates that the best-fitting model (KM+exp) cannot reproduce the observed data, and that none of the considered models is adequate for these two data sets.

### Hidden Population Structure and Population Decline in the Data Set Lee 2015

In our analysis, we focused on local data sets to control for the confounding effect of complex population dynamics and population structure. However, in one case (Lee 2015), it is possible that some degree of population structure was still present. Lee 2015 is a data set sampled from an epidemic in Inuit villages in Nunavik, QC, Canada (Lee, Radomski, Proulx, Levade, et al. 2015). Lee, Radomski, Proulx, Levade, et al. (2015) showed that transmission of MTB among patients was more frequent within a village than between villages, and that related strains tended to be present in the same village. This was supported by the reconstructed phylogenetic tree, which showed three clades separating at the root that could represent distinct subpopulations (supplementary fig. 14, Supplementary Material online; see also figure 2 in Lee, Radomski, Proulx, Levade, et al. 2015). These data suggest the existence of some degree of geographic population structure. Therefore, we tested whether this might influence the results of our model selection. To do this, we ran two analyses:

1. We repeated the ABC-RF analysis on three subsets of Lee 2015, which represent the three main clades described above (supplementary fig. 14, Supplementary Material online). Under the assumptions that the separate branches of the phylogeny reflect different subpopulations, and that migration does not alter the coalescent rates within the subpopulations, the genealogy of each subclade should then follow one of the coalescent models that we are fitting. We found that BETA was the best-fitting model for two of the subclades, whereas Dirac was the best-fitting model for the third (table 3). The posterior predictive checks showed that the best model could reproduce the data of these three subsets (supplementary figs. 15–17, Supplementary Material online). However, the posterior probabilities were low compared with the complete data set, and the misclassification probabilities were larger. This was probably due to the smaller sample size of the individual subsets compared with the full data set (table 3).
2. We performed an additional model selection analysis between three competing models, BETA, Dirac and a third scenario, in which we modeled a structured population with migration and with standard offspring distribution and exponential growth (KM+exp; see Materials and Methods for details). Also in this case, BETA resulted to be the most likely model (table 3). Overall, our findings indicate that it is unlikely that the MMC signal in the Nunavik MTB population is an artifact caused by population structure.

**Table 3.** **Results of Model Selection for the Complete Lee** 2015 **Data Set, and for the Three Major Subclades Separately.**

Data Set	No. of Strains	Selected Model	OOB Error Rate (misclassification % as MMC)^a^	Posterior Probability	Second Best-Fitting Model
Lee 2015	147	BETA	6.8% (2.6%)	96.0%	Dirac
Lee 2015 Clade A	61	Dirac	20.2% (10.0%)	82.2%	BETA
Lee 2015 Clade B	36	BETA	16.6% (6.8%)	64.4%	KM
Lee 2015 Clade C	49	BETA	12.8% (5.0%)	78.6%	Dirac
Lee 2015 Pop. structure^b^	147	BETA	8.5% (6.9%)	94.4%	Dirac
Lee 2015 Pop. decline^c^	147	BETA	3.4 % (3.0%)	99.9%	Pop. decline

Note.—The shaded row represents the results of the standard analysis on the full data set.

a The OOB error rate is the probability that a simulation is misclassified as coming from any other model class, between parentheses we report the probability that a simulation generated with KM or KM+exp is misclassified as MMC (BETA or Dirac).

b Model selection among BETA, Dirac, and KM with structure.

c Model selection among BETA and KM with population decline.

Structured populations have similar genealogies to populations that are shrinking in size (forward in time), with many lineages coalescing close to the tips of the genealogy. In their original publication, Lee, Radomski, Proulx, Levade, et al. (2015) used the BSP (Drummond et al. 2005) to reconstruct the fluctuations in population size of the Nunavik population and found evidence for a slow population decline. Here, we are not interested in whether the inferred population decline is genuine or caused by unaccounted population structure; we only want to assess whether a decline in population size could bias our analysis. We repeated the ABC-RF model selection among two models: BETA and KM with population decline (see Materials and Methods for details). Again, we found that BETA was the best-fitting model (table 3), indicating that our results for this data set are unlikely to be an artifact caused by population decline.

### Serial Sampling

One limitation of our analysis is that it assumes that all samples were collected at the same time (synchronous sampling). Generally, MTB strains are sampled from the sputum of patients, which is collected when they first present for diagnosis. All data sets that resulted in an MMC as best-fitting model included samples obtained over extended periods of time (serial sampling), corresponding to between ∼8% and ∼100% of the estimated tree age (supplementary table 3, Supplementary Material online).

We investigated whether, at least in principle, the violation of the assumption of synchronous sampling could bias the results of the ABC analysis performed above, and whether the better fit of MMC could be an artifact due to such violation. To do this, we generated simulated data assuming serial sampling and performed model selection on the simulated data assuming synchronous sampling (see Materials and Methods). As this analysis depends on assumptions about the sample size, the genetic diversity, and the sampling times, we used the settings (sample size, observed generalized Watterson’s estimator as scaled mutation rate, and the real years of isolation) of three of the observed data sets, which differed in these characteristics (Eldholm 2015, Lee 2015, and Roetzer 2013).

**Fig. 1.**
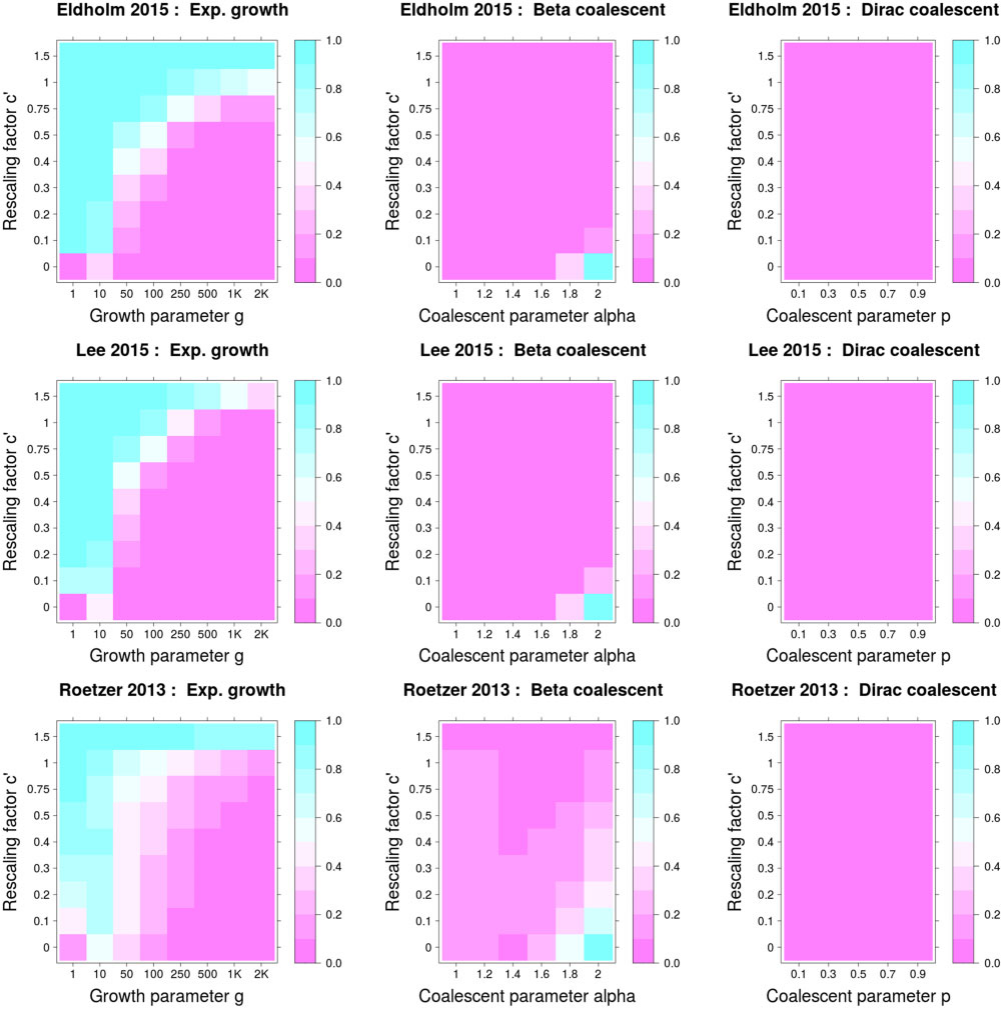
Proportion of model misidentification for serial simulations when model selection was performed via ABC using ultrametric tree models. Misclassification probabilities are shown as a function of *c*′ (the proportion of the genealogy corresponding to the time period in which samples are collected, that is, the period of sampling spans a time period *c*′·*h*, where *h* is the expected height of the genealogy without serial sampling), and of the parameter of the coalescent models. Misclassification was measured as follows: 1) for simulations from serially sampled Kingman’s coalescent with exponential growth as being misidentified as either Beta or Dirac (first column); and 2) for simulations from serially sampled Beta or Dirac coalescents as being misidentified as Kingman’s coalescent with or without exponential growth (second and third columns).

We found that data simulated under KM+exp can be misclassified as BETA or Dirac if we do not account for serial sampling. Specifically, this was true for extended sampling periods compared with the expected height of the genealogy (on the coalescent time scale), and for low growth rates (fig. 1). Similarly to model selection, not accounting for serial sampling affected the estimation of the growth rate parameter, and this effect was greater for large sampling periods and low growth rates (supplementary fig. 18, Supplementary Material online).

It is difficult to relate these results to the observed data sets because we do not know the scaling factor between coalescent time and real time (and therefore cannot estimate the value of *c* in fig. 1, see Materials and Methods). However, for six of the eight data sets that resulted in an MMC as best-fitting model, we estimated large growth rates (*g* ≥ 1,000) under the KM+exp model (supplementary table 2, Supplementary Material online), indicating that serial sampling is unlikely to affect the results of model selection in these cases (fig. 1 and supplementary fig. 18, Supplementary Material online).

Nevertheless, we adopted a complementary approach, in which we drastically reduced serial sampling by subsampling only strains that were isolated in a single year. As small data sets have lower discriminatory power, for this analysis, we selected the four data sets with the highest genetic diversity (data sets with more than 200 polymorphic positions: Eldholm 2015, Lee 2015, Folkvardsen 2017, and Bainomugisa 2018) among the ones for which the sampling times were available. For each data set, we repeated the ABC analysis on the largest possible subset of strains that were sampled in a single year (supplementary table 1, Supplementary Material online, and table 4). We found that all subsets had lower posterior probabilities and higher misclassification errors compared with the full data sets, most likely because of the smaller sample size. KM+exp was the best-fitting model for two subsets, and Dirac and BETA were the best-fitting model for one subset each. For the data sets Eldholm 2015 and Folkvardsen 2017, the second and third most sampled years had a similar number of strains compared with the most sampled year. Therefore, we extended the analysis to these four additional subsets, which all resulted in BETA as the best-fitting model (table 4). We also noticed that in the subset of Lee 2015 (sampled 2012), all strains but one belonged to one of the three clades discussed above (clade A; supplementary fig. 14, Supplementary Material online). We suspected that this analysis was influenced by population structure and we repeated it excluding the single strain not belonging to clade A. Again, we found that Dirac was the best-fitting model (table 4 and supplementary table 2, Supplementary Material online).

**Table 4.** Results of Model Selection for the Temporal Subsets.

Data Set^a^	No. of Strains	Selected Model	OOB Error Rate (misclassification % as MMC)^b^	Posterior Probability	Second Best-Fitting Model
Eldholm 2015	248	BETA	5.4% (2.1%)	96.6%	KM+exp
Eldholm 2015 (1998)	34	KM+exp	20.9% (9.5%)	75.9%	BETA
Eldholm 2015 (2001)	31	BETA	18.3% (7.7%)	79.9%	Dirac
Eldholm 2015 (2003)	32	BETA	18.3% (7.8%)	83.0%	KM+exp
Lee 2015	147	BETA	6.8% (2.6%)	96.0%	Dirac
Lee 2015 (2012)	45	Dirac	15.8% (6.5%)	96.3%	BETA
Lee 2015 (2012) Clade A	44	Dirac	22.7% (11.3%)	84.5%	BETA
Bainomugisa 2018	81	BETA	8.9% (3.3%)	97.6%	KM+exp
Bainomugisa 2018 (2014)	56	BETA	11.3% (4.2%)	94.9%	KM+exp
Folkvardsen 2017	702	BETA	4.0% (1.4%)	97.8%	KM +exp
Folkvardsen 2017 (2009)	53	BETA	14.0% (5.8%)	85.1%	KM+exp
Folkvardsen 2017 (2010)	64	KM+exp	13.5% (5.7%)	83.8%	BETA
Folkvardsen 2017 (2012)	52	BETA	15.1 % (6.4%)	96.8%	KM

Note.—Shaded rows contain the results for the full data sets.

a Between parentheses we report the year in which the strains were sampled (only for temporal subsets).

b The OOB error rate is the probability that a simulation is misclassified as coming from any other model class, between parenthesis we report the probability that a simulation generated with KM or KM+exp was misclassified as MMC (BETA or Dirac).

We performed posterior predictive checks for all subsets and found that in all cases the best-fitting model could reproduce the observed data (supplementary figs. 19–27, Supplementary Material online).

Overall, these findings indicate that not accounting for serial sampling can indeed bias the results of model selection in favor of MMC models. However, this was unlikely to affect data sets fitting to large growth rates (six out of eight). Additionally, seven of the nine subsets in which we minimized the serial sampling to one single year resulted in an MMC as best-fitting model.

### Sensitivity to the Choice of Prior Distributions

An important aspect of Bayesian analyses is to test whether the results are robust to different priors and model assumptions. Therefore, we performed a set of analyses investigating the sensitivity of the results of the ABC to changes to the prior distribution of the growth rate (*g*) for KM+exp, of the parameter α for BETA, and of the scaled mutation rate (θ) for all models. These analyses are reported in supplementary appendix 1, Supplementary Material online. Overall, we performed four additional ABC analyses testing different prior combinations, and for two of them we also tested the data sets obtained with the 75% threshold on the SNP call (supplementary table 2 and supplementary figs. 28–35, Supplementary Material online). We found that in 89% of cases, the results of model selection did not change compared with the main analysis presented above. The data sets that were sensitive to the prior choice were mostly the ones with low sample sizes, low number of polymorphisms, low posterior probabilities, and large error rates, further highlighting that the results of small data sets should be taken with some caution.

### Modeling Skewed Offspring Distribution and Variable Population Size

So far, we considered only multiple merger models with constant effective population size. However, for many of the outbreak analyzed, it is reasonable to expect that the effective population size was growing (forward in time). We therefore performed a further model selection between the best-fitting model resulted from the analysis described above, and two additional model classes: BETA with exponential population growth (BETA+exp) and Dirac with exponential population growth (Dirac+exp; see Materials and Methods for details).

Overall, we found that 22 of the 23 data sets (including subsets) resulted in an MMC (with or without growth) as best-fitting model. For 11 data sets we found that the best-fitting model was an MMC with exponential growth, suggesting that these populations were indeed growing in size (supplementary table 2, Supplementary Material online). However, for some data sets the posterior probabilities were low, indicating that different models fitted the data similarly well.

### Skewed Offspring Distribution Can Bias Demographic Inference with the BSP

**Fig. 2.**
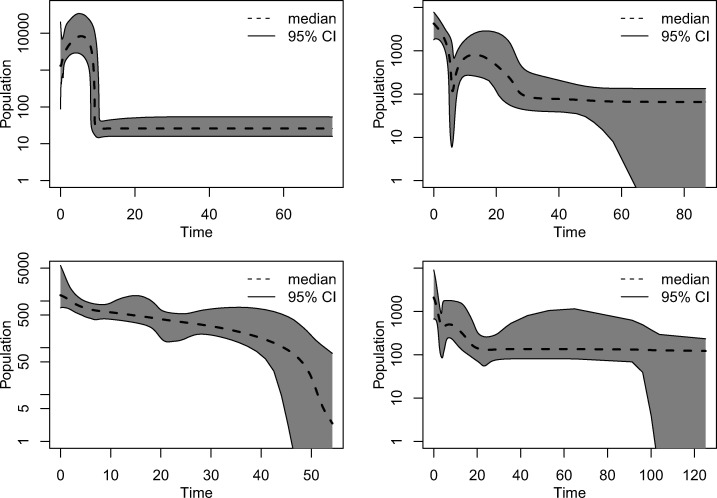
Four examples of BSP obtained from data simulated under a Beta coalescent with constant effective population size, and α = 1 (top-left), α = 1.25 (top-right and bottom-left), and α = 1.5 (bottom-right). On the *y*-axis the inferred effective population size, on the *x*-axis the time in years before sampling. The plots for all 50 simulations are reported in supplementary figures 36–40, Supplementary Material online.

To reconstruct the past demographic history of MTB and other organisms, many studies use nonparametric approaches such as the BSP (Heled and Drummond 2008). In the last few years (since 2013), at least 16 studies applied the BSP to MTB data sets (cited in the Introduction). It was shown before that the BSP can be biased by unaccounted population structure (Heller et al. 2013), recombination, and nonrandom sampling (Lapierre et al. 2016). Hence, we next assessed the impact of skewed offspring distribution on demographic reconstruction with the BSP. To do this, we simulated 50 data sets under the BETA coalescent with constant population size. We used different values of α corresponding to the range of values estimated for the observed MTB data sets (α = 0.5, 0.75, 1, 1.25, 1.5; ten replicates each, see Materials and Methods). We then performed an extended BSP analysis on the simulated data using BEAST2 (Bouckaert et al. 2019). For 49 of the 50 simulated data sets we found that the 95% highest posterior distribution interval of the number of population size changes did not include zero, thus rejecting the constant population size model (Heled and Drummond 2008). The inferred skyline plots showed different patterns of fluctuation of the effective population size (fig. 2 and supplementary figs. 36–40, Supplementary Material online). These results demonstrate that skewed offspring distribution alone can bias the outcome of the BSP, leading to the inference of complex population dynamics that are entirely due to the violation of the assumption on the offspring distribution.

## Discussion

As for many other organisms, demographic inference based on Kingman’s coalescent has become an important tool to study the evolution and epidemiology of MTB. As always when performing statistical inference, the results of these analyses depend on the assumptions of the model.

Our results showed that, when studying MTB local population and outbreaks, models that do not allow for skewed offspring distribution on the coalescent timescale (Kingman) had consistently worse fit compared with MMC and can lead to the inference of false fluctuations of past effective population size.

### Better Fit of MMC Models Is Robust to Possible Confounders

We tested the robustness of our results by changing different aspects of the analysis, including one of the main SNP call parameters and the choice of prior distributions in the ABC (supplementary appendix 1, Supplementary Material online). We performed eight different model selection analyses on all 23 data sets (including subsets), and invariably found that a large majority of the data sets resulted in an MMC as best-fitting model (supplementary table 2, Supplementary Material online). When we included MMC models with exponential growth in an additional ABC analysis, only one data set (Stucki 2016) resulted in Kingman as the best coalescent type. However, for this data set, the posterior predictive checks could not reproduce the observed genetic diversity, indicating that none of the models used was adequate (supplementary fig. 13, Supplementary Material online). Although our results are robust to different settings, it is important to discuss some of the assumptions on which they are based, in particular regarding 1) sampling bias, 2) population structure, and 3) serial sampling.

1. One factor that could bias the results of demographic inference is sampling bias (Lapierre et al. 2016). The majority of the data sets considered in this study is composed of strains sampled from (nearly) all known tuberculosis cases caused by a certain phylogenetic clade (Roetzer et al. 2013; Lee, Radomski, Proulx, Levade, et al. 2015; Stucki et al. 2015; Bjorn-Mortensen et al. 2016; Eldholm et al. 2016) or by a random subset of them (Folkvardsen et al. 2017). Therefore, these data sets should not be strongly affected by sampling bias.
2. In our analyses, we assumed that the data originated from single panmictic populations. For clonal organisms, or when only a single locus is available, recognizing population structure from genetic data is challenging, because clusters of genetically similar individuals can occur also in a panmictic population (at a single locus). Therefore, to avoid the effect of unaccounted population structure, we chose data sets from single outbreaks and local populations in restricted geographic regions. However, we cannot completely exclude that for some data sets some level of geographic structure was present, and this might have affected the results of our analyses.

For one data set where a prior analysis suggested some degree of population structure (Lee, Radomski, Proulx, Levade, et al. 2015), we found that including a model with population subdivision and migration, or subsampling the potential subpopulations, resulted again in an MMC as the best-fitting model, indicating that population structure is unlikely to bias the results of this analysis.

(3)Serial sampling could also affect the results of model selection. All the considered models assume a common sampling time for all strains, which is almost never the case for MTB data sets. Due to the relatively short generation time of MTB compared with the sampling period, this may likely correspond to serial sampling on the coalescent time scale. Under serial sampling on the coalescent time scale, our simulations of several scenarios mimicking three of our data sets revealed that Kingman’s genealogies under exponential growth can be misidentified as MMC, while inferring a true MMC is not affected. The misclassification probability was higher when the sampling window spanned a large part of the genealogical history of the sample but dropped considerably under strong exponential growth (fig 1 and supplementary figs. 18, 28, and 29, supplementary appendix 1, Supplementary Material online). For six of the eight full data sets that resulted in an MMC as best model, the fitted growth parameter under KM+exp was 1,000 or higher; and therefore, it is unlikely that the serial sampling influenced the results of model selection for these data sets. Additionally, when we subsampled strains from a single year from four data sets, thus minimizing the effect of serial sampling, seven of the nine subsets resulted in an MMC model (table 4). For most data sets, the better fit of MMC is unlikely to be an artifact caused by serial sampling. Nevertheless, it is possible that serial sampling influenced some of these results. Obtaining truly synchronous samples is essentially impossible for MTB, and reducing the sampling time to 1 year or few months could still not be enough to avoid serial sampling on the coalescent time scale. Thus, to overcome the limitation of assuming synchronous sampling, we encourage future studies to develop MMC models that explicitly consider the time of sampling. Such a model is proposed in Hoscheit and Pybus (2019) as an extension of the Beta coalescent but without an explicit mechanism to convert real time units into coalescent time units.

Our result that the Beta coalescent is generally fitting better to outbreaks of MTB is in contrast with the use of the Dirac coalescent as the model for within-host MTB genealogies in Morales-Arce et al. (2020). Although within-host and between-host dynamics are surely different, it would be interesting to test whether the Beta coalescent also fits better within-host data. A hint that this could be the case is that the strength of multiple mergers (the coalescent parameter ψ) estimated in Morales-Arce et al. (2020; figure 3) was very low.

### What Are the Processes Generating Multiple Merger Genealogies in MTB?

At least three different biological processes could lead to MMC genealogies in MTB: 1) repeated bottlenecks at transmission between hosts, 2) superspreaders, and 3) rapid recurrent selection induced by the immune system, and/or by antibiotic treatment.

To formally test which of these three processes (if any) is generating the MMC genealogies in outbreaks of MTB, we would need to have an explicit population model for each of these scenarios and for their combinations. Additionally, for each population model, we would need to know the corresponding coalescent process. If these three mechanisms (or their combinations) lead to different coalescent processes, we could then use the observed data to test the different hypotheses.

However this information is only partially available:

1. Although the Beta coalescent has been proposed as genealogy model for populations with recurrent strong bottlenecks (Tellier and Lemaire 2014), rigorous mathematical modeling predicted different coalescent processes for extreme bottlenecks, which allow for simultaneous multiple mergers (Birkner et al. 2009; Casanova et al. 2019). In any case, these models are not appropriate for MTB outbreaks, because they model bottlenecks affecting the whole population. Transmission bottlenecks in an MTB population can be thought as multiple serial founder events. To model such processes, one would need to account for both intrahost dynamics within multiple hosts and between-hosts transmission. Such a model is currently not available, and it is not clear to which coalescent process it would lead. It is possible that the Beta or the Dirac coalescent captures well the main features of the genealogies generated by such a mechanism. However, without an explicit model a formal test is impossible.
2. The Beta coalescent has also been proposed to model the genealogies of epidemics with superspreaders. A simulation study showed that the parameter α contains information about the degree of superspreading, with lower values of α corresponding to higher level of superspreading (and larger multiple merger events; Hoscheit and Pybus 2019). However, also in this case, an explicit population model is missing. Thus, we cannot formally test whether MMC genealogies in MTB outbreaks are due to superspreaders.
3. The genealogies of populations evolving under recurrent rapid positive selection are described by the BSZ coalescent (Bolthausen and Sznitman 1998; Brunet and Derrida 2013; Neher and Hallatschek 2013), which correspond to the Beta coalescent with α = 1. Therefore, by estimating the value of α from the data, we can partially test whether rapid selection is the single factor causing the MMC signal in MTB outbreaks. More precisely, if the 95% credibility interval (CI) of the posterior distribution of α does not include one, we can exclude that rapid selection is the only factor involved.

For five of the data sets that resulted in an MMC as the best-fitting model, the 95% CI of α did not include one (supplementary appendix 1; supplementary figs. 33–35, Supplementary Material online). These results suggest that for these data sets we can exclude recurrent rapid positive selection as the only process leading to multiple merger genealogies; whereas for many others, the data fitted well with the BSZ. For instance, two data sets, Eldholm 2015 and Bainomugisa 2018, represent outbreaks of drug-resistant clones which acquired several additional drug resistance mutations during the outbreak (Eldholm et al. 2015; Bainomugisa et al. 2018). It is possible that strains with novel drug resistance mutations were advantaged, resulting in recurrent positive selection. This scenario would lead to the BSZ (corresponding to α = 1), and indeed the estimates of α encompassed 1 for both data sets. In this analysis, we used the BSZ coalescent as a null model for ongoing constant selection pressure, as also proposed in Neher and Hallatschek (2013). This coalescent model arises in several models of ongoing selection in a rapidly adapting fixed-size population, where the constant size is maintained by viability selection against the least fit individuals (Berestycki et al. 2013; Brunet and Derrida 2013; Neher and Hallatschek 2013). In these models, multiple mergers occur when an individual becomes much fitter than the rest of the population. However, we want to stress that there are alternative MMC models that correspond to different selection scenarios, such as a model with a modified fitness distribution (Huillet 2014), models considering the effect of several beneficial mutations (Desai et al. 2013; Huillet 2014; Schweinsberg 2017), or hitchhiking of a neutral locus with a single beneficial mutant (Gillespie 2000). In addition, selection could also act in combination with other (selectively neutral) sources of multiple mergers, as discussed in Der et al. (2011, 2012) or Etheridge et al. (2010).

Because of the lack of explicit models, we cannot test the exact biological process or processes generating multiple merger genealogies in MTB. The better fit of MMC models only indicates that one or more processes leading to skewed offspring distribution (on the coalescent time scale) play an important role in shaping the diversity of MTB populations. Moreover, our estimates of α, the inference of the best-fitting MMC models (Beta or Dirac), and whether the best-fitting model included an exponential growth component, differed distinctively across data sets. This suggests that different processes, or different magnitudes of the same process, produced multiple merger genealogies in different populations. For example, two of the data sets that had an estimate of α lower than 1 (corresponding to larger multiple merger events, see supplementary appendix 1, Supplementary Material online) were sampled from different outbreaks with important superspreading events (Stucki 2015, Lee 2015 Clade A). Stucki 2015 is a data set representing an outbreak characterized by one key patient that had two distinct disease episodes, separated by 3 years, both of which resulted in a large number of secondary infections (Stucki et al. 2015). The data set Lee 2015 Clade A is a sample of an outbreak occurred in an Inuit village in Quebec (Canada), in this case two patients were responsible for 75% of all secondary infections (Lee, Radomski, Proulx, Manry, et al. 2015; Lee et al. 2020). These data strongly suggest that for these data sets the mechanism generating large multiple mergers was the superspreading behavior of one or few patients. We found only one additional data sets that resulted in α lower than 1, and interestingly it was also an outbreak among Inuit villages in East Greenland (Bjorn-Mortensen et al. 2016). Lee, Radomski, Proulx, Manry, et al. (2015) and Lee et al. (2020) found that all secondary cases of the Canadian outbreak visited the same local community gathering houses. It is possible that such social gatherings played a similar role also in the spread of the Greenlandic outbreak leading to large multiple merger events.

A further observation is that that the magnitude of multiple mergers might also change through time. For example, the temporal subsets of Folkvardsen 2017 fitted values of α around 1, or larger than 1, depending on the year of sampling (supplementary fig. 34, Supplementary Material online).

Additional processes might also generate multiple merger genealogies. For instance, MTB infections can remain latent for several years and reactivate under favorable circumstances (e.g., immunosuppression due to age, or HIV coinfection), although these cases might be less common than previously thought (Behr et al. 2018). It is known that stochastic exit from dormancy can lead to heavy-tailed offspring distributions, with bacteria exiting dormancy earlier having an extremely high reproductive success (Wright and Vetsigian 2019). Also in this case, mathematical modeling will be necessary to investigate whether this mechanism can affect the genealogies of MTB and to identify the coalescent type that would result from such process.

Finally, MMC could also arise from the violation of another assumption of the Kingman coalescent, specifically when the sample size is on the same order of magnitude (or larger) than the effective population size (Wakeley and Takahashi 2003). This could be relevant for outbreaks in which the effective population size is small because all strains descend from a very recent common ancestor.

### Modeling MTB Genealogies

A population model for MTB should include host-to-host transmission, intrahost evolution, superspreaders, serial sampling, latency, population size changes, and the potential selective pressure caused by the host immune system and/or by the antibiotic treatment; although some of these factors might not influence strongly the genetic diversity when modeled in combination with other mechanisms. This might very well result in a different multiple merger model compared with the ones that we employed. Such a model could also close the following implicit modeling gap in applying MMC to MTB and to bacteria in general. Mathematically, MMC processes have been introduced as approximations (with changed time scale) of the genealogy in underlying discrete population reproduction models, so called Cannings models (e.g., Möhle and Sagitov 2001). The underlying population models feature many offspring of a single individual per generation (e.g., Schweinsberg 2003; Eldon and Wakeley 2006; Desai et al. 2013). However, bacteria replicate through binary fission. Although such population models are not applicable directly to bacteria, the underlying mathematical theory only needs to guarantee that the mergers within a single time point on the coalescent time scale follow a certain probability distribution, such that similar models can be defined, in which the large offspring number of one individual per generation is spread over multiple generations (Möhle and Sagitov 2001).

## Conclusions

In this study, we investigated whether the Kingman’s assumption of low variance of reproductive success is violated in MTB populations, and whether demographic inference with Kingman as null model could lead to artifacts due to model misspecification. We found that MTB genealogies are indeed affected by skewed offspring distribution and that this can significantly bias the results of demographic inference, resulting in spurious past population dynamics. Potentially, these results can be extended to other obligate pathogens with similar life histories.

Further research is needed to develop an explicit population model for MTB. This would help to identify the biological mechanisms leading to multiple merger genealogies, and the most appropriate genealogy model for MTB populations. In the meantime, we encourage researchers to be extremely cautious when interpreting the results of demographic inference of MTB data sets based on the Kingman coalescent.

## Materials and Methods

### Data Set Selection

We searched the literature for WGS studies of outbreaks or local populations of MTB*.* We selected local data sets to avoid as much as possible geographic population structure and sampling biases that could influence the analysis. We identified 11 data sets: eight outbreaks and three clades with a restricted geographical range (the inferred phylogenies for all data sets are reported in supplementary figs. 41–51, Supplementary Material online):

- Roetzer et al. 2013*:*lineage 4 outbreak in Hamburg, Germany (61 strains, 74 polymorphic positions).
- Comas et al. 2015*:*lineage 7 strains sampled in Ethiopia. Lineage 7 is a rare human-adapted lineage endemic to Ethiopia and perhaps also to neighboring countries, only few genomes are available and most of them are included in this data set (21 strains, 1,334 polymorphic positions).
- Eldholm et al. 2015*:*lineage 4 multidrug-resistant outbreak in Buenos Aires, Argentina (248 strains, 497 polymorphic positions).
- Lee, Radomski, Proulx, Levade, et al. 2015*:*lineage 4 outbreak in 11 Inuit villages in Nunavik, QC, Canada. We considered only the major sublineage Mj, a second smaller outbreaks of an unrelated sublineage (Mn) was excluded (147 strains, 454 polymorphic positions).
- Stucki et al. 2015*:*lineage 4 outbreak in Bern, Switzerland (60 strains, 128 polymorphic positions).
- Bjorn-Mortensen et al. 2016*:*lineage 4 outbreak in Greenland. To minimize the potential effect of population structure we considered only the major cluster GC4, because the other clusters represent independent outbreaks belonging to other sublineages (121 strains 128 polymorphic positions).
- Stucki et al. 2016*:*sublineage L4.6.1/Uganda, belonging to lineage 4. This sublineage is endemic to central African countries (175 strains, 6,264 polymorphic positions).
- Eldholm et al. 2016*:*lineage 2 outbreak in Oslo, Norway. From the data set of the original publication, we excluded all strains that did not belong to the Oslo outbreak (25 strains, 17 polymorphic positions).
- Folkvardsen et al. 2017*:* large lineage 4 outbreak in Copenhagen, Denmark (702 strains 514 polymorphic positions).
- Shitikov et al. 2017*:*W148 outbreak belonging to lineage 2, this clade has also been named B, B0, CC2, East European 2, and ECDC0002 (176 strains, 1,164 polymorphic positions).
- Bainomugisa et al. 2018*:*lineage 2 multidrug-resistant outbreak on a small island (Daru) in Papua New Guinea. From the data set of the original publication, we excluded all the strains that did not belong to the Daru outbreak (81 strains, 401 polymorphic positions).

### Bioinformatic Pipeline

For all samples Illumina reads were trimmed with Trimmomatic v0.33 (SLIDINGWINDOW:5:20,ILLUMINACLIP:{adapter}:2:30:10) (Bolger et al. 2014). Reads shorter than 20 bp were excluded for the downstream analysis. Overlapping paired-end reads were then merged with SeqPrep (overlap size = 15; https://github.com/jstjohn/SeqPrep; last accessed July 26, 2020). The resulting reads were mapped to the reconstructed MTB complex ancestral sequence (Comas et al. 2013) with BWA v0.7.12 (mem algorithm; Li and Durbin 2009). Duplicate reads were marked by the MarkDuplicates module of Picard v 2.1.1 (https://github.com/broadinstitute/picard; last accessed July 26, 2020). The RealignerTargetCreator and IndelRealigner modules of GATK v.3.4.0 (McKenna et al. 2010) were used to perform local realignment of reads around Indels. Reads with alignment score lower than (0.93*read_length)-(read_length*4*0.07)) were excluded: This corresponds to more than seven mismatches per 100 bp.

SNPs were called with Samtools v1.2 mpileup (Li 2011) and VarScan v2.4.1 (Koboldt et al. 2012) using the following thresholds: minimum mapping quality of 20, minimum base quality at a position of 20, and minimum read depth at a position of 7× minimum percentage of reads supporting a call 90%.

Genomes were excluded if they had 1) an average coverage <20×, 2) more than 50% of their SNPs excluded due to the strand bias filter, 3) more than 50% of their SNPs having a percentage of reads supporting the call between 10% and 90%, or 4) contained SNPs that belonged to different MTB lineages, as this indicates that a mix of genomes was sequenced. Because missing data can significantly impact population genetic inference, we further excluded all strains that had less SNP calls than (average − (2 * SD)) of the respective data set (calculated after all previous filtering steps). The filters described above were applied to all data sets with one exception: In the Comas 2015 data set most strains failed the strand bias filter, therefore this filter was not applied.

To test the robustness of our results, we performed an additional SNP call, in which we used a 75% threshold on the minimum proportion of reads supporting a call.

The single vcf was merged with the CombineVariant module of GATK v.3.4.0 (McKenna et al. 2010), and the genotype field was edited to make it haploid (0/0 => 0; 1/1 => 1; 0/1 and 1/0 => .). Vcftools 0.1.14 (Danecek et al. 2011) was used to extract variable positions excluding predefined repetitive regions (Comas et al. 2013) and excluding position with missing data.

The variable positions were converted in a multi fasta file including the reconstructed ancestral sequence on which the mapping was performed.

A phylogenetic tree based on the resulting variable positions was built with RaxML 8.2.11 (Stamatakis 2014) using a GTRCAT model and the -V option.

To identify the MRCA of each data set, the tree was rooted using the reconstructed ancestral sequence of the MTB complex as published in Comas et al. (2013), which is also the genome reference sequence used for the mapping. PAML4 (baseml; Yang 2007) was used to reconstruct the ancestral sequence of each data set. For all data sets, the sequence accuracy (the marginal probability of the reconstructed sequence) of the MRCA was larger than 0.999.

For each data set, all polymorphic positions for all strains and their reconstructed ancestor were then collected in fasta files. The data (obtained with both the 90% and 75% threshold) are available together with the ABC pipeline at https://github.com/fabianHOH/mmc_R_gendiv/tree/master/MTB_MMC_repo (last accessed July 26, 2020).

### Model Selection and Parameter Estimation

For model selection and parameter estimation, we used a random forest-based ABC approach (Pudlo et al. 2016; Raynal et al. 2019).

We selected between Kingman’s n-coalescent (KM), Kingman’s n-coalescent with exponential growth (KM+exp), Beta coalescent (BETA), and Dirac coalescent (Dirac). For each data set, we collected the genetic polymorphisms identified with the bioinformatic analysis and calculated a set of 24 summary statistics following the recommendations from Freund and Siri-Jégousse (2020), Scenario 3: the (0.1, 0.2, 0.3, 0.4, 0.5, 0.6, 0.7, 0.8, 0.9) quantiles of the mutant allele frequency spectrum, the (0.1, 0.3, 0.5, 0.7, 0.9) quantiles of the pairwise Hamming distances, the (0.1, 0.3, 0.5, 0.7, 0.9) quantiles of the minimal observable clade sizes of each sequence, the number of segregating sites, the nucleotide diversity and the mean, standard deviation and harmonic mean of the minimal observable clade sizes. For each model we performed 125,000 simulations of a sample of size *n* where *n* is the number of individuals in the data set, drawing the scaled mutation rate from a binomial distribution on log-equally spaced discrete θ spanning 1 order of magnitude around the Watterson estimator (θ_obs_), that is, 11 steps in [θ_obs_/5,5θ_obs_], as in Freund and Siri-Jégousse (2020). The Watterson estimator is calculated as 2 *s*/*E*(*L*), where *s* is the number of mutations observed in the data set and *E*(*L*) is theexpected length of the genealogy. For KM+exp we drew the value of the exponential growth rate (*g*) from a uniform distribution on [0.5, 5,000] except for the data sets Bainomugisa 2018, Bjorn-Mortensen 2016, Eldholm 2015, Stucki 2015, Lee 2015 (sampled in 2012), and Folkvardsen 2017, where we used a uniform distribution on [0.5, 20,000]. Note that this is a growth rate for a coalescent within a diploid population, and values should be halved for interpretation in a haploid setting. The choice of wider ranges was based on preliminary analyses of the data with narrower prior distributions that showed a posterior distribution of *g* skewed at the upper end. For comparison, we also used an alternative setting with log-uniform priors on these ranges (supplementary appendix 1, Supplementary Material online). For BETA and Dirac, we drew the value of the free parameters α and ψ from a uniform distribution on [1, 2] and [0, 1], respectively. Additionally, we performed an alternative analysis in which we drew the value of α from a uniform prior distribution on [0, 2] (supplementary appendix 1, Supplementary Material online). Note that although BSZ is theoretically included in BETA as α = 1, it will not be chosen as a parameter because we use a continuously distributed prior. To further assess whether BSZ is a well-fitting model, we alternatively employed a spike and slab type prior, that is, we replaced 1% of all parameters drawn from the continuous uniform prior for BETA with α = 1. We used this in an additional ABC analysis on BETA with α from [0, 2], the log prior on the growth rate for KM+exp, and the standard setting for Dirac (supplementary appendix 1, Supplementary Material online).

Simulations were performed in R as described in Freund and Siri-Jégousse (2020), and the code is available at https://github.com/fabianHOH/mmc_R_gendiv (last accessed July 26, 2020).

As described in Pudlo et al. (2016), we performed model selection via ABC using a random forest of decision trees, using the R package abcrf (Pudlo et al. 2016). We drew 1,000 bootstrap samples of size 100,000 from the simulations and then constructed decision trees based on decision nodes of the form *S* > *t*, where *S* is one of the summary statistics used. For each node, *S* and *t* are chosen so that the bootstrap sample is divided as well as possible in sets coming from the same of the four model classes (minimal Gini impurity). Nodes are added to the tree until all simulations of the bootstrap samples are sorted into sets from the same model class. Misclassification is measured by the OOB error, that is, the proportion of decision trees for each simulation that sorts it into a wrong model class, averaged over simulations and, for the overall OOB error, model classes.

For parameter estimation within a model class, we followed Raynal et al. (2019). Here, the decision (regression) trees are constructed analogously, only *S* and *t* are chosen so that the parameters of the simulations have similar values in both sets divided by the node. This is achieved by minimizing the *L*^2^ loss, that is, minimizing, for the two sets divided by the node, the *L*^2^ distances of the simulation parameter to the mean parameter in the set. Nodes are added until all simulations sorted into one leaf have the same parameter or there are <5 simulations allocated to the leaf.

The observed data are then assigned to the model class where the majority of decision trees for model selection assigns it, and its posterior parameter distribution is given by the distribution of the weighted average parameter of the allocated leaf across all trees in the (regression) random forest (see Raynal et al. 2019, Sections 2.3.2 and 2.3.3). The posterior probability for model selection is computed as a machine-learning estimate of classifying the model class correctly, which includes another regression tree. (See Pudlo et al. [2016] for details, a summary can be found in Appendix A.2 in Freund and Siri-Jégousse [2020]).

### Misclassification Probabilities

The misclassification probabilities were calculated as follows. After building the random forest, all simulated data sets were assigned to one of the models based on a random forest composed only of trees built from bootstrap samples not including this simulation (so that the data were not used to produce the decision trees). As the true model is known, we can easily calculate the proportion of trees that classify this simulation in a wrong model class. The OOB error rate for a model class is the proportion of misclassified simulations over all simulations from the model class. The mean OOB error is the average of OOB errors across model classes. More informative error rates can also be calculated, for example, the proportion of simulations from a bifurcating model that are classified as a multiple merger.

### Posterior Predictive Checks

To assess whether the best-fitting model could reproduce the observed data, we performed posterior predictive checks. We simulated 10,000 sets of summary statistics under the best-fitting model (using the median of the posterior growth rate or of the multiple-merger coalescent parameter, obtained from the main analysis, Analysis 1 in supplementary table 2, Supplementary Material online) and compared them graphically with the value of the statistics observed in each data set. As scaled mutation rate, we used the generalized Watterson estimate 2 *s*/*E*(*L*), where *s* is the number of mutations observed in the data set and *E*(*L*) is the expected length of all branches for the best-fitting coalescent model.

### Population Structure and Declining Population Size for the Data Set Lee 2015

To assess the effect of population structure in the data set Lee 2015, we simulated samples under Kingman’s n-coalescent with population structure. From the phylogenetic tree (supplementary fig. 14, Supplementary Material online), we identified four different clades with sizes 61, 36, 49, and 1. We then assumed these to be sampled from different subpopulations of equal size in an island model with scaled symmetric migration. We performed coalescent simulations under a structured (Kingman) coalescent with exponential growth. We used a discrete uniform prior on {0, 2, 4, … , 5,000} for growth rates and additionally drew the scaled migration rate *m* (in units of 4Nm*, where m* is the migration rate in the discrete island model) from the uniform discrete distribution {0.25, 0.5, 1, 2, 3}. We approximated Watterson’s estimator for a specific choice of parameters by replacing the expected total length of the coalescent by the mean total length from 10,000 coalescent simulations with these parameters. This approximation comes with an increased computational load compared with our standard approach, which in turn led us to the discretization of the prior described above.

For generating samples under Kingman’s n-coalescent with exponential decline, we had to slightly change the simulation procedure using ms. As population decline may lead to coalescent times to large too simulate, we fixed the maximal population size in the past to 1,000 times the present population size. Then, given an exponential growth rate *g* < 0, the decline starts at time log(1,000)/(−*g*) (in coalescent time units backwards in time from time of sampling) and continues until the sampling time.

To compute Watterson’s estimator in this scenario for any *g*, we need the expected total length of the coalescent tree. Instead of computing it analytically, we recorded the total coalescent tree length of 10,000 simulations under the model and used their mean as an approximation of the expected total branch length.

As parameters for exponential decline, we use exponential growth rates drawn uniformly from {−250, −200, −150, −100, −50, −25, −10}, again we used a discrete prior distribution because Watterson’s estimator was too costly to approximate for a continuous range.

For both exponential decline and population structure, we ran the ABC-RF analysis as for all other data sets. Simulations were produced with Hudson’s ms (Hudson 2002) as implemented in the R package phyclust.

### Accounting for Serial Sampling

Following Hoscheit and Pybus (2019), we add serial sampling to the MMC and to Kingman’s coalescent with exponential growth simply by stopping the coalescent at times (on the coalescent time scale) where further individuals are sampled. Then, we start a new (independent) coalescent tree that has rates and waiting times as the nonserial coalescent (multiple merger or with growth) started in the last state of the stopped coalescent plus adding one block with a single individual for each individual sampled at this time. A R implementation is available at https://github.com/fabianHOH/mmc_R_gendiv/tree/master/MTB_MMC_repo (last accessed July 26, 2020).

A problem with this approach is that one needs the scaling factor between coalescent time and real time. Although estimation procedures coming from phylogenetics are available in the case of Kingman’s coalescent (e.g., Drummond and Rodrigo 2000), they cannot be applied directly to the case of MMC. Additionally, a brute force search for appropriate scaling on top of our models is computationally unfeasible with the ABC approach that we adopted in this study.

Hence, we assessed, for different fixed scaling factors, how strong the effect of ignoring serial sampling in the models is. We considered the setting of Eldholm 2015 (*n* = 248, *s* = 497, where *n* is the sample size and *s* is the number of mutations), Lee 2015 (*n* = 147, *s* = 454), and Roetzer 2013 (*n* = 61, *s* = 74). We used the real dates of the serial sampling for these data sets and we performed serial coalescent simulations as described above. We used different time (re)scaling factors *c*, such as *c* determines the time ct at which an individual sampled at real time −*t* (0 corresponds to the latest sampling time) is added as a new lineage to the coalescent tree (so ct is in coalescent time units). Here, we assessed *c* by setting the earliest sampling time (highest t) to a fraction *c*′ ≥ 0 of the expected height of the coalescent tree if there was no serial sampling (so keeping all other parameters, but assuming *c* = 0). For each *c*′ in {0, 0.1, 0.2, 0.3, 0.4, 0.5, 0.75, 1,1.5}, we simulated 1,000 simulations under each parameter set (*g* in {1, 10, 50, 100, 250, 500, 1,000, 2,000}, α in {1, 1.2, 1.4, 1.6, 1.8, 2}, ψ in {0.1, 0.3, 0.5, 0.7, 0.9}) and then performed ABC model selection for each simulation (as described above), recording how often the serial coalescent simulations were sorted to which nonserial model class. We also reported the quality of parameter estimation for the growth rate or coalescent parameter by measuring the (absolute) distances of the estimated parameter to the parameter used for the simulation (supplementary figs. 18 and 29, Supplementary Material online).

### MMC with Exponential Growth

We define Beta coalescents with exponential growth as limit processes of modified Moran models with variable population sizes, as described in Corollary 1 and Equation (10) in Freund (2020). This means that we take a Beta(2 − α, α) coalescent, but change the time scale with the function *t* → (*g*α)^−1^(exp(*g*α*t*) − 1), where *g* is the exponential growth rate; that is, the time changed coalescent at time *t* corresponds to the original coalscent at time (*g*α)^−1^(exp(*g*α*t*) − 1). For Dirac coalescents, we consider the approach as described in Matuszewski et al. (2018), which adds exponential growth to the modified Moran models with skewed offspring distributions from Eldon and Wakeley (2006). This results in a Dirac coalescent whose time scale is changed by the function *t* → (1.5*g*)^−1^(exp(1.5*gt*) − 1), regardless of the Dirac coalescent parameter (we choose γ = 1.5 in the prelimit modified Moran models).

We again approximate the Watterson estimator by approximating the total expected length of the coalescent tree with the mean over 1,000 simulations of the chosen Dirac coalescent with exponential growth. Due to the computational load, this led us to use a discrete prior, a uniform distribution on both the coalescent parameter and the growth parameter.

For both models, the growth rates were uniformly chosen as exp(*g*) from ten equidistant steps *g* between 0 and log(5,000) (including both values), in other words we used a discretized log uniform prior on growth rates. The coalescent parameter was similarly chosen from ten equidistant steps between 1 and 1.9 for BETA (so 1, 1.1, …, 1.9) and between 0.05 and 0.95 for Dirac.

We performed a model section between these two models and the best-fitting model class resulted from the main analysis (Analysis 1 in supplementary table 2, Supplementary Material online). We performed 125,000 simulations (reduced to 80,000 for Stucki 2016 and Folkvardsen 2017 due to large computation times). As for all other scenarios, we drew the scaled mutation rate from a binomial distribution on log-equally spaced discrete θ spanning 1 order of magnitude around the Watterson estimator (θ_obs_), and then performed the ABC analysis as described above.

### Bayesian Skyline Plots

We simulated data under the BETA coalescent with different values of alpha spanning the range of estimates obtained from the observed data (α = 0.5, α = 0.75, α = 1, α = 1.25, α = 1.5; ten simulations for each value of α). For this analysis, we used the settings corresponding to a medium sized data set such as Eldholm 2015 (*n* = 250, *s* = 500, θ set to the generalized Watterson’s estimate 2*s*/*E*(*L*)). The simulated data sets are lists of mutations and their states (derived or ancestral) for all individuals. We transformed the simulated data in sequence data by randomly assigning nucleotides (A, T, C, or G) to ancestral and derived mutations, drawing them from the empirical nucleotide frequency distribution of the data set Eldholm 2015. For all 50 simulated data sets, we ran an extended Bayesian skyline analysis (Heled and Drummond 2008) with BEAST 2.5.0 (Bouckaert et al. 2019).

We assumed a strict clock, with clock rate equal to 5 × 10^−8^ nucleotide changes per site per year, a value that falls in the range of possible evolutionary rates for MTB (Menardo et al. 2019). Importantly assuming that a different evolutionary rate would result in a different time scale, but it would not affect the demographic reconstruction. We assumed the GTR (general time reversible) substitution model, and a 1/X [0–100,000] prior on the mean of the distribution of population sizes. For each data set we ran two runs of 400 million generations, discarded the first 40 million generation as burn-in and combined the two runs. For all data sets, the effective sample sizes of the posterior distribution and of the number of changes in population size (sum(indicators.alltrees)) were larger than 180 (and in most cases larger than 200). The plots were produced with the plotEBSP R script available here (https://www.beast2.org/tutorials/; last accessed July 26, 2020). The simulated data and one example of the BEAST input (xml) files are available at https://github.com/fabianHOH/mmc_R_gendiv/tree/master/MTB_MMC_repo (last accessed July 26, 2020).

## Supplementary Material

Supplementary data are available at *Molecular Biology and Evolution* online.

## Supplementary Material

msaa179_Supplementary_Data

## Notes

^†^These authors contributed equally to this work.
